# Innovations in Geropsychology Training and Workforce Development: Graduate School through Post-Licensure

**DOI:** 10.1093/geroni/igaf122.777

**Published:** 2025-12-31

**Authors:** Michelle Mlinac, Erin Kube, Patricia Bamonti

## Abstract

Following the 2021 Building Bridges Conference on geropsychology workforce development, this symposium highlights novel innovations in geropsychology training. In 2023, the specialty updated its training taxonomy, formal guidance on the recommended offerings for each level of opportunity (“exposure” through “major area of study”) from graduate school through post-licensure levels of development. The first presentation reviews results from a recent survey of the landscape of geropsychology training following the COVID pandemic, highlighting issues of burnout among geropsychology trainees and faculty. The second presentation offers a new competency framework outlining the knowledge and skills recommended to provide clinical supervision to geropsychology trainees. The third presentation shares the results of a 2025 survey of geropsychologists’ learning needs around behavioral sleep medicine, to create continuing professional education to bolster geropsychologists’ clinical skills in this area. The fourth presentation reviews a pilot program to broaden the workforce and foster exposure to clinical practice with older adults via monthly consultation for generalist mental health providers at the masters and doctoral levels. The fifth presentation describes the process of board-certification for geropsychologists with the aim of increasing the numbers of those who deliver specialty care to the public. It also describes the development of a clinical consultation model to support a pathway for practicing psychologists who were not specialty trained in geropsychology in graduate school or fellowship to become eligible for board-certification at the post-licensure level. The innovations in this symposium may interest other geriatric clinical professions looking to meet similar training and workforce needs.

